# Improved target detection method for space-based optoelectronic systems

**DOI:** 10.1038/s41598-024-51717-0

**Published:** 2024-01-21

**Authors:** Rui Zhu, Qiang Fu, Nan Liu, Feng Zhao, Guanyu Wen, Yingchao Li, Huilin Jiang

**Affiliations:** 1 Jilin Provincial Key Laboratory of Space Optoelectronics Technology, Changchun University of Science and Technology, Changchun, China; 2 Institute of Optoelectronic Engineering, Changchun University of Science and Technology, Changchun, China; 3 Changchun Observatory, National Astronomical Observatories, Chinese Academy of Sciences, Changchun, China

**Keywords:** Astronomy and planetary science, Optics and photonics

## Abstract

The detection of faint and small targets by space-based surveillance systems is difficult owing to the long distances, low energies, high speeds, high false alarm rates, and low algorithmic efficiencies involved in the process. To improve space object detection and help prevent collisions with critical facilities such as satellites, this study proposes an improved method for the detection of faint and small space-based targets. The proposed method consists of two components: star atlas preprocessing and space-based target detection. The star atlas preprocessing step applies multi-exposure image pyramidal weighted fusion to the original image containing the faint and small space-based target. After obtaining the image pyramidal weighted fusion result atlas, the algorithm employs threshold segmentation to improve the overall image clarity, highlight image details, and provide additional information for target detection. The detection of targets partially relies on the local symmetry of the image. Accordingly, a diffusion function describing the local symmetry is established to precisely locate stars by measuring the symmetry factor in a small area surrounding each pixel in the star atlas. This effectively removes the background stars while retaining high-definition and high-contrast images. The efficacy of the algorithm is validated using simulated datasets consisting of space-based and real images. The results demonstrate that the proposed technique improves the applicability of the multistage hypothesis testing (MHT) method in the context of a complex space environment, thus improving the performance of the space-based electro-optical detection system to better catalogue, identify, and track space targets.

## Introduction

Orbital surveillance systems that are used to monitor space-based targets play an important role in the security of space assets, are fundamental to national strategic facilities, and represent an important direction for the future development of space situational awareness technologies. They are also cutting-edge technology in the field of space exploration, which is of strategic importance for the successful execution of national space missions and the maintenance of national security systems. Surveillance of space-based targets is primarily performed via radar detection, photoelectric detection, and other techniques. Optoelectronic detection technology offers the benefits of high-resolution images, large detection distances, compact system sizes, and low costs; these favourable properties facilitate the detection of many objects in space and meet the requirements of space-based target detection^1,2^.

Optical imaging is the most common way for space-based surveillance systems to acquire information on the space environment. In optical images, space-based targets appear as point-like targets without any information on their sizes, structures, and textures. Therefore, space-based target detection is often considered a problem of detecting faint and small targets in complex backgrounds. A major obstacle that prevents space-based surveillance systems from quickly and accurately detecting space-based targets is the lack of a priori information, such as the target trajectory, direction of motion, position in the image, and image characteristics of the target and the background stars, which is compounded by the influence of image noise^3^. Space-based target detection technology primarily includes two components: star atlas preprocessing and faint target detection technology.

Star atlas preprocessing is defined as the processing of images prior to the detection and positioning of spatial targets in the image. Due to stray radiation and detector interference, space-based optical systems often capture images with a high level of noise, which reduces the image quality and hinders the extraction of the target characteristics. Image preprocessing primarily utilises transform domain methods and spatial domain methods. In transform domain denoising methods, the frequency domain low-pass filtering^4^ and wavelet transform^5,6^ techniques are extensively used. Frequency domain low-pass filtering employs a two-dimensional discrete Fourier transform to convert the image information to the frequency domain. Subsequently, because noise typically behaves as a high-frequency component in the frequency domain, low-pass filtering is applied in the frequency domain to filter out the noise signal. Prominent spatial domain denoising techniques include median^7^, mean^8^, Wiener^9^, and morphological^10^ filtering. Despite this wide range of available techniques, none of them include the properties of the star atlas itself, which has resulted in unsatisfactory image processing outcomes.

In the field of space-based target detection, scholars have proposed methods such as multi-frame time series projection^11^, trajectory identification^12,13^, matching correlation^14^, and hypothesis testing^15–18^ by investigating the discrepancy between the space-based target to be detected and the background of the star atlas. However, space-based background noise is complex, variable and diverse, and consists of four main aspects: (1) interference from stray light due to light scattering and reflection, etc.; (2) interference from inhomogeneous and random noise due to various factors; (3) interference from the large number of stars present on target detection; and (4) difficulty in accurately distinguishing between spatial targets in the star atlas because of the similarity between their forms and grayscale values and the noise. These aspects lead to the fact that the methods in the literature mentioned above are often not effective in denoising space-based images, which results in the loss of target information or the generation of false targets.

**Figure 1:**
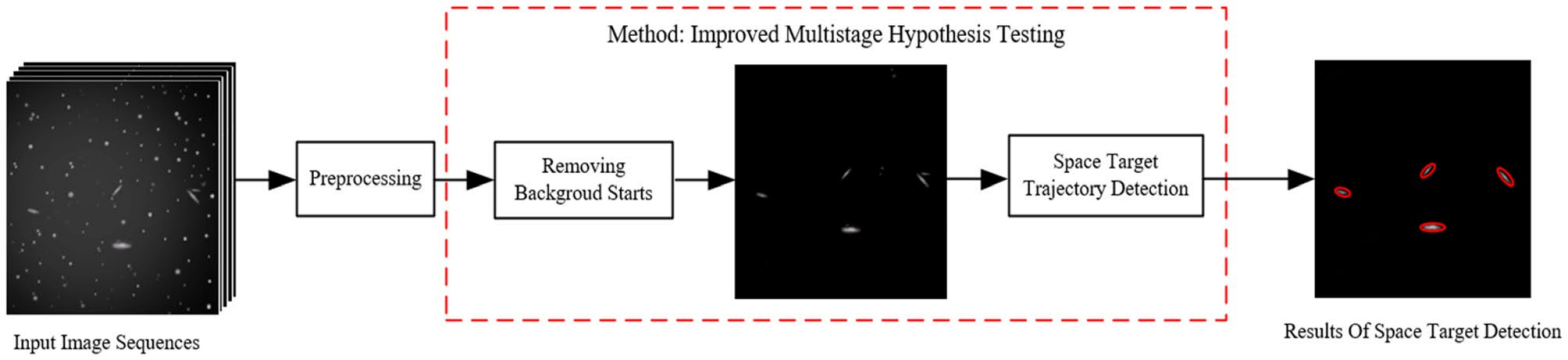
Flow chart of the detection of faint and small space-based targets.

To overcome these difficulties, this study proposes a modified multi-exposure image pyramidal fusion method based on the algorithm developed by T. Mertens^19^. The proposed method calculates the pixel weights of the fused image based on three indicators: contrast, two-dimensional entropy, and exposure goodness of the sequence image before fusion. The saturation function of the fusion coefficients in the original algorithm is replaced by an entropy information function. The improved algorithm can neglect the influence of illumination on the image when extracting the edges as well as improve the sharpness, contrast, and greyscale variance of the edge contours of the star atlas. This produces image fusion results that are more natural and richer in detail. For the space-based detection of faint and small targets, this study presents an improved multistage hypothesis testing (MHT) method. The traditional MHT algorithm constructs the trajectories of a large number of candidate targets in a sequence of space-based images in a tree structure, and it prunes each frame in the image sequence via hypothesis testing conditions, resulting in excellent detection performance. However, an excessive number of stars in the image can lead the MHT algorithm to require a significant computational load. By invoking a diffusion function to measure the local asymmetry within a small region around each pixel in the star atlas to pinpoint the stars, the proposed method can effectively remove background stars, decrease the complexity of the algorithm, and improve the performance of the space-based detection of faint and small targets while retaining high-definition and high-contrast images. The flow of the proposed algorithm is illustrated in Fig. 1.

The rest of this paper is organised as follows. "Star atlas preprocessing" describes the star atlas preprocessing step. "Exposure quality" explains the improved MHT target detection method. In “Experimental evaluation”, the effectiveness of the improved algorithm is validated by simulated and real star atlases. Finally, “Conclusion” contains the conclusions of this study.

## Star atlas preprocessing

Faint targets with low signal-to-noise ratios occupy only a few pixels, and their successful detection is highly sensitive to background clutter and noise. Therefore, it is difficult to detect the targets directly; this necessitates the preprocessing of the star atlas to suppress image noise and improve the efficiency of the detection algorithm. Star atlas preprocessing involves the utilisation of median filtering and multi-exposure image pyramidal fusion techniques. We first perform star atlas preprocessing instead of removing the stars in the background of the image is that star atlas preprocessing can improve the overall clarity of the star atlas and highlight the details of the image, which has the advantage of providing a good foundation for the next star removal and target detection, improving the success rate of target detection and reducing false alarms.

The median filtering technique is used to estimate the nonuniform background of each image, and the original image is subtracted from the background image in order to remove the nonuniformity of the image. The median filter is not used for stars, and its main function is to remove noise such as pretzels or non-uniformity in the star atlas. For targets, there is no loss of low SNR targets using median filtering, because whether or not low SNR targets are lost depends on the size of the filtering window, and in this paper a smaller window (3 × 3) is used to better preserve the details of the targets. The median filtering formula is

1 $$g\left( {x,y} \right) = \mathop {median}\limits_{(m,n) \in N(x,y)} f(m,n)$$

where $f\left( {m,n} \right)$ represents the initial image data,$g\left( {x,y} \right)$ is the filtered image data, and $N\left( {x,y} \right)$ is a two-dimensional template for a window for selecting elements and performing median calculations.

### Improved multi-exposure image fusion

In complex space-based backgrounds, images can appear to have excessively high contrast between light and dark areas, resulting in overexposed and saturated bright areas throughout the image, underexposed dark areas, and missing edge information and texture detail. To address these issues, T. Mertens proposed a pyramid-based image fusion algorithm, which fuses the three functions of image contrast, colour saturation, and exposure quality; this enables the acquisition of the pixel weights of the fused image and generates superior fusion outcomes^19^. This study aimed to make this algorithm more applicable to the detection of faint and small targets in space-based environments. We employed a two-dimensional entropy information function instead of the saturation function of the fusion coefficients in the original algorithm. The effect of illumination on the image was neglected when extracting edges, resulting in a more natural and detailed fusion result, as well as an improvement in the sharpness, contrast, and greyscale variance of the edge contours of the star atlas.

### (1) Image contrast

The original multi-exposure image is Laplace-filtered, assuming that if the grey value of a point is greater than its surroundings after filtering, the point is a bright spot with dark surroundings; otherwise, it is a dark spot with bright surroundings. The contrast coefficient is used to evaluate the value of that point relative to its surroundings. Therefore, the absolute value of the pixel at that location is acquired, and the resulting absolute value response coefficient reflects information about the contrast of the image at each pixel. The weight coefficients are produced by computing the changes in the image edge, turning the image into a greyscale one, and normalising the pixels in the image to the interval^1^. Calculating the second-order gradient of the image provides a more comprehensive description of the image contrast information. The Laplace operator is

2 $$\nabla^{2} g\left( {x,y} \right) = \frac{{\partial^{2} g\left( {x,y} \right)}}{{\partial^{2} x^{2} }} + \frac{{\partial^{2} g\left( {x,y} \right)}}{{\partial y^{2} }}$$

and the convolutional template can be derived from the Laplace operator as

3 $$h = \left[ {\begin{array}{*{20}c} 0 & 1 & 0 \\ 1 & { - 4} & 1 \\ 0 & 1 & 0 \\ \end{array} } \right]$$

### (2) Two-dimensional entropy

**Figure 2:**
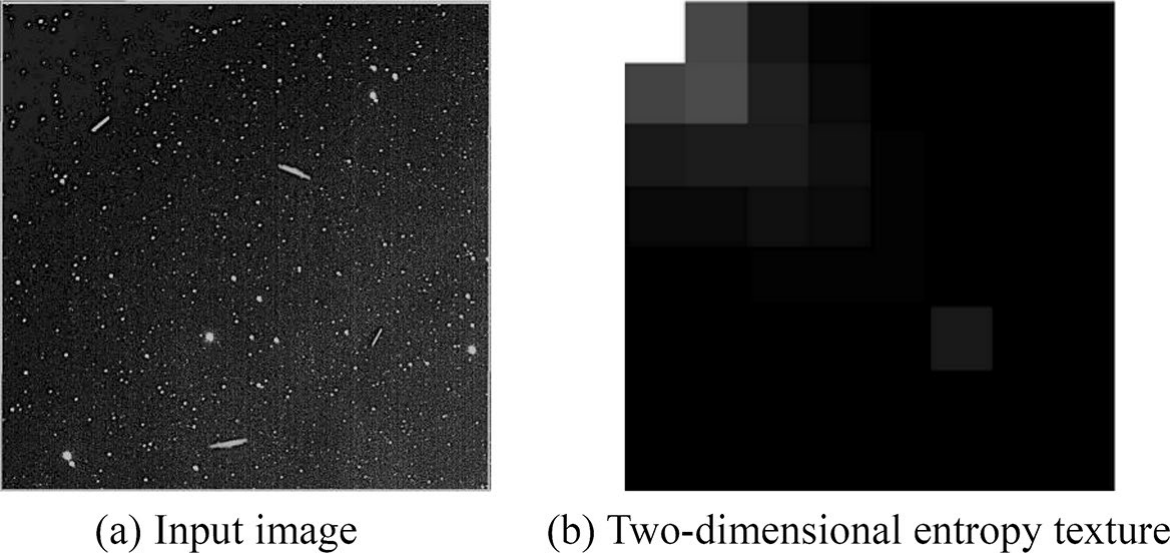
2D entropy information image.

Entropy is typically used as a method to evaluate the focus and to assess the amount of information contained in an image; the higher the entropy, the clearer the image. The one-dimensional entropy of an image may describe the aggregated features of the greyscale distribution of the image, but not its spatial qualities. To characterise these spatial properties, the two-dimensional entropy of an image may be introduced to represent the combined qualities of the greyscale information of the pixels and the greyscale distribution in the neighbourhood of each pixel. The expression for the two-dimensional entropy *E* required for the assessment of a greyscale image is

4 $$E = - \sum\limits_{i = 0}^{255} {\sum\limits_{j = 0}^{255} {G_{i,j} \log_{2} } } G_{i.j}$$

where *i* represents the pixel's grey value, *j* represents the neighbourhood’s grey mean, and $G_{i,j}$ represents the combined properties of the pixel's grey value and the grey distribution of the surrounding pixels.

5 $$G_{i,j} = \frac{c(i,j)}{{WH}}$$

where $c\left( {i,j} \right)$ is the number of occurrences of the feature binary $\left( {i,j} \right)$; W and H denote the image size. Figure 2 shows the 2D entropy information image obtained by grey-level co-occurrence matrix, and the two-dimensional entropy information value is 0.2024.

### (3) Exposure quality

The image pixels are normalised, typically, the greyscale value is to 0.5, set the value to 0.5 because this makes the brightness in the fusion result more balanced and retains more detail. Details in lower exposure images will not be too dark and details in higher exposure images will not be too bright. This achieves a more natural and balanced result, and the pixel weighting at a given exposure is indicated by $W\left( {x,y} \right)$, which is defined as

6 $$W\left( {x,y} \right) = \frac{{ - \left[ {{\text{V}} \left( {x,y} \right) - 0.5} \right]^{2} }}{{2\sigma^{2} }}\begin{array}{*{20}c} {} & {0 \le {\text{V}} \left( {x,y} \right) \le 1,\sigma = 0.2} \\ \end{array}$$

where ${\text{V}} \left( {x,y} \right)$ represents the normalized pixel value, pixel normalization can be achieved by dividing all pixel values by the maximum pixel value, which is 255. because normalizing the pixels is more convenient for calculating the weight value of the exposure factor and does not affect anything else.

The image pixels are chunked into a 3 $\times$ 3 matrix, with the pixel centroids representing the amount of information in the matrix. The main reason for segmenting image pixels into a 3 × 3 matrix is to obtain sufficient exposure range and detail information. The 3 × 3 exposure range is chosen to cover relatively low brightness, medium brightness and relatively high brightness. This ensures that more details and dynamic range are captured during the fusion process to produce images with high dynamic range.

3 × 3 and 5 × 5 each have their own advantages over each other, with 3 × 3 allowing for more detailed treatment of localized details in the image and 5 × 5 allowing for better treatment of smooth areas. Whether to use 3 × 3 or 5 × 5 depends on the content of the image data and the desired effect. In this paper, the choice of 3 × 3 exposure range is a balance in multi-exposure pyramid fusion that provides sufficient dynamic range and detail information while maintaining reasonable computational complexity.

The total weight is calculated using

7 $$w^{k} \left( {x,y} \right) = \left[ {\left( {C^{k} \left( {x,y} \right) + a} \right)} \right]^{wc} \cdot \left[ {\left( {E^{k} \left( {x,y} \right) + b} \right)} \right]^{ws} \cdot \left[ {\left( {W^{k} \left( {x,y} \right) + c} \right)} \right]^{we}$$

where $k$ is the sequence number of the image. *C*, *E*, and *W* represent the image's contrast, entropy, and exposure level of the image, respectively; $a = b = c = 1$; $C^{k} \left( {x,y} \right)$ is the contrast weighting; $E^{k} \left( {x,y} \right)$ is the entropy weighting; $W^{k} \left( {x,y} \right)$ is the exposure weighting factor; and $wc$, $ws$, and $we$ are the weighing factors (the number 1 is often used).

The total weight values are normalised to

8 $$\tilde{w}^{k} (x,y) = \left[ {\sum\limits_{k = 1}^{N} {w^{k} } (x,y)} \right]^{ - 1} w^{k} (x,y)$$

where N is the number of input images. As *N* increases, the output of the final weighting atlas used to determine the coefficients becomes more precise.

Applying Eq. (8) to successive images captured with various exposure times results in

9 $$R\left( {x,y} \right) = \sum\limits_{k = 1}^{N} {\tilde{w}^{k} } (x,y)I^{k} \left( {x,y} \right)$$

where $R\left( {x,y} \right)$ is the image output and $I^{k} \left( {x,y} \right)$ is the kth input image.

The algorithmic outline of this section is shown below.
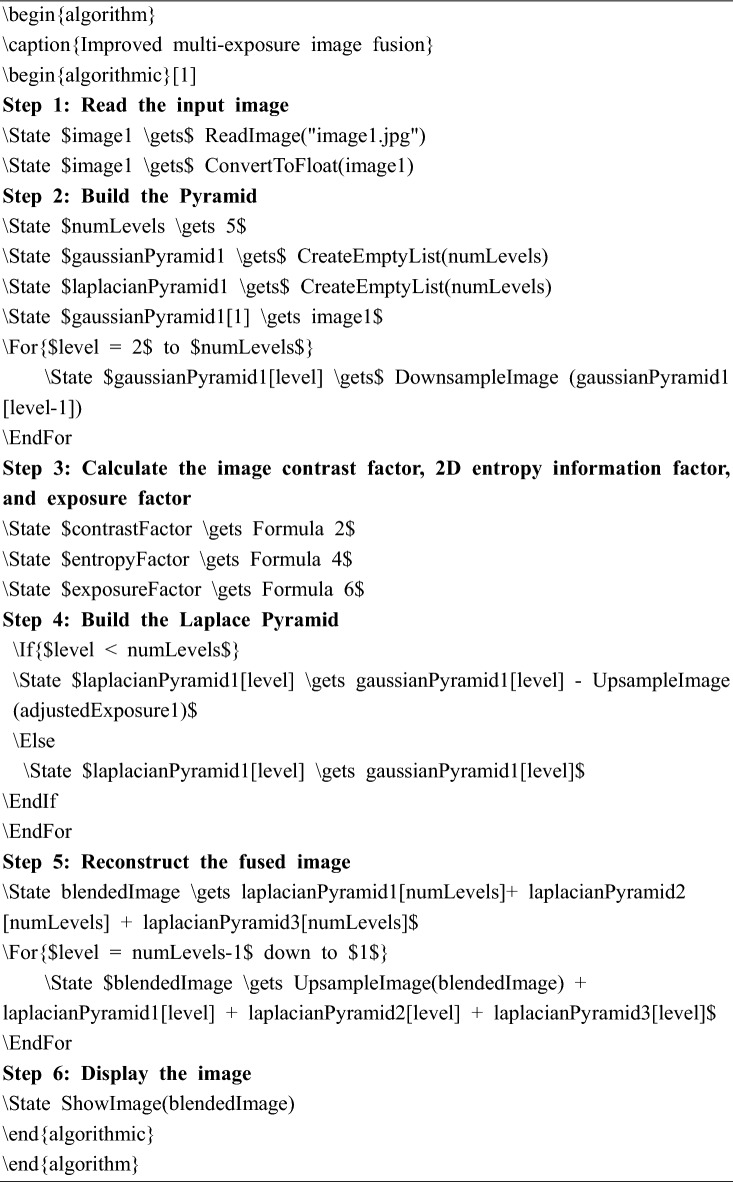


### Threshold segmentation

In space-based image processing, weak spatial targets may be misclassified as part of the background if the image is segmented immediately using mean and standard deviation-based thresholds without suppressing stellar and other spatial targets. To address this challenge, a threshold segmentation approach that combines global and local threshold segmentation is adopted according to the following stages.

1. The global thresholding $T_{G}$ is an adaptive threshold segmentation for the whole image and is defined as
  10 $$T_{G} = \mu + \alpha \sigma$$
  where $\mu$ is the average image background noise,$\sigma$ is the standard deviation, and $\alpha$ is a constant .
2. After global threshold segmentation, the image is characterised by
  11 $$B_{G} (x,y) = \left\{ {\begin{array}{*{20}c} 1 & {R(x,y) > T_{G} } \\ 0 & {R(x,y) \le T_{G} } \\ \end{array} } \right.$$
3. Local threshold segmentation separates the image into $N \times N$ areas and determines the threshold $T_{Li}$ for each region, which is defined as
  12 $$T_{Li} = \mu_{i} + \alpha \sigma_{i}$$
  where $\sigma_{i}$ is the standard deviation of the ith partition area, and $i$ = 1,2,3, …, $N \times N$_,_ and $\mu_{i}$ is the mean of the first partition region.
4. The results of local threshold segmentation are
  13 $$B_{L} (x,y) = \left\{ {\begin{array}{*{20}c} 1 & {R(x,y) > T_{Li} } \\ 0 & {R(x,y) \le T_{Li} } \\ \end{array} } \right.$$
5. For efficient identification of spatial targets with a low signal-to-noise ratio, the same value of $\alpha$($\alpha = 2$) is used in Eqs. (10) and (12), the main reason for setting $\alpha$ to 2 is to balance the brightness and contrast of the image. When $\alpha = 2$, the threshold is set to twice the mean value of the image. This means that pixels lower than the mean value will be considered as background and pixels higher than the mean value will be considered as foreground.

Different values of $\alpha$ lead to different thresholding choices and segmentation results. A smaller $\alpha$ value (e.g. 1) will result in a threshold close to the image mean, which may result in too many pixels being classified as foreground, resulting in a higher false alarm rate. A larger value of $\alpha$ (e.g. 3) moves the threshold away from the image mean, which may result in more pixels being classified as background, thus producing a higher false alarm rate. Therefore, choosing $\alpha = 2$ is a compromise that balances the effect of foreground and background segmentation to some extent.

The final candidate points for overall threshold segmentation are derived as follows:

14 $$B\left( {x,y} \right) = B_{G} \left( {x,y} \right) \cdot B_{L} \left( {x,y} \right)$$

**Figure 3:**
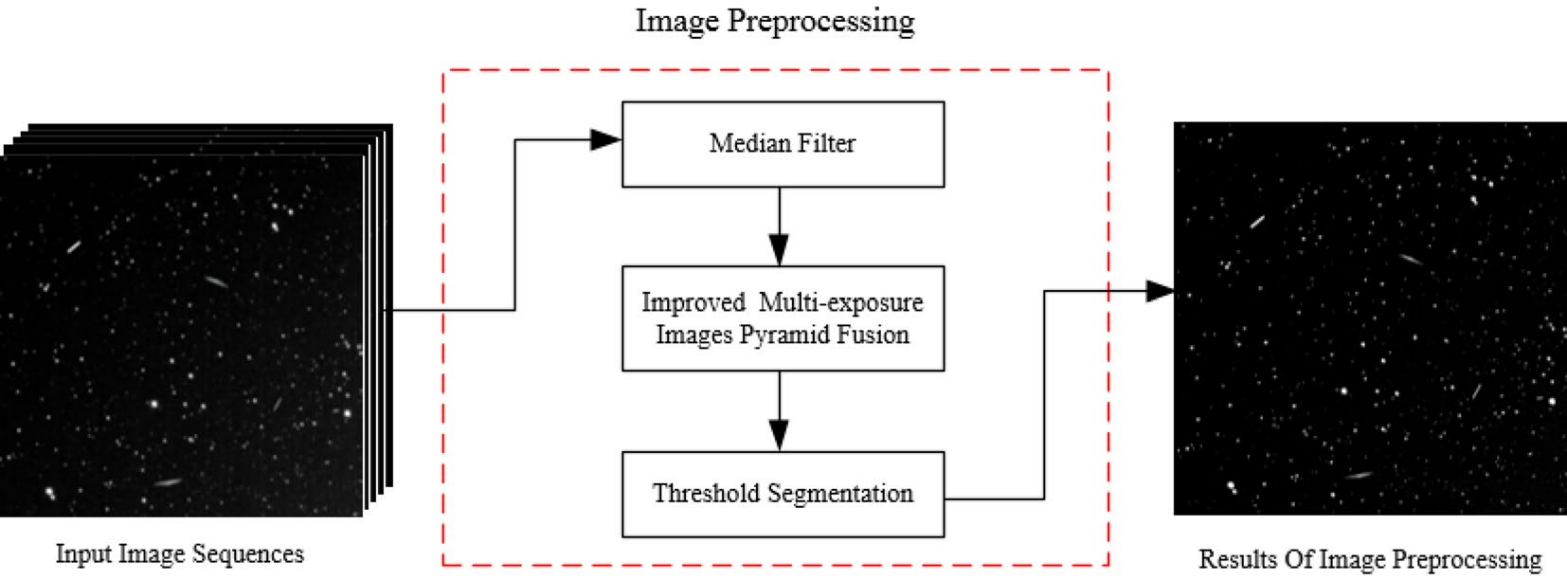
Image preprocessing flow chart.

Figure 3 depicts the image preprocessing sequence. First, median filtering is used to denoise the star map. Second, image quality enhancement is performed using an improved multi-exposure image pyramid fusion method. Finally, threshold segmentation separates the target from the star atlas background to simplify subsequent analysis and processing.

## Improved target detection with multistage hypothesis testing

**Figure 4:**
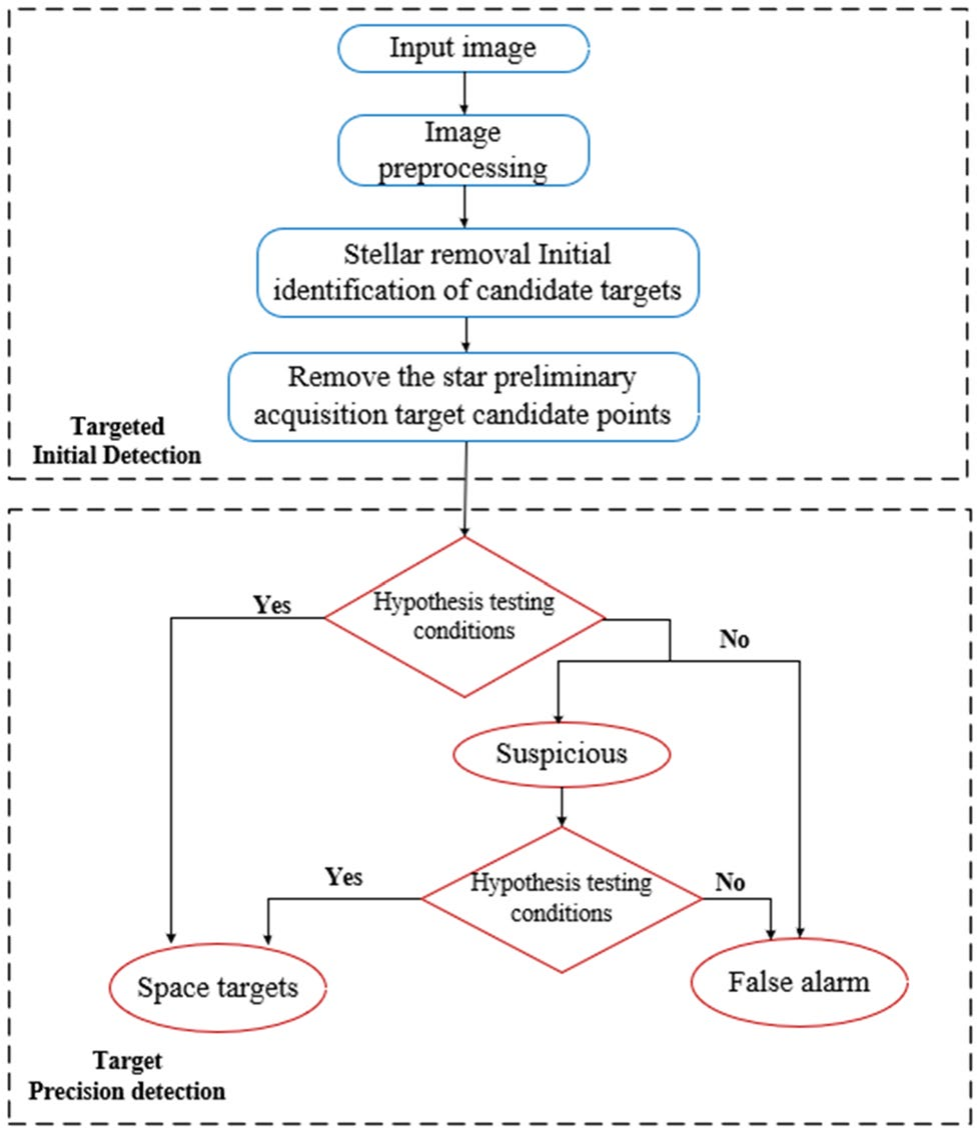
The overall flowchart of the algorithm.

Blostein's^15^ MHT technique correlates each frame using a tree structure, adds the grey values of each frame, and thresholds them appropriately to filter the nodes at each level of the discriminant tree and to determine the trajectory of the target. The method is effective for detecting faint targets with unknown positions and velocities. However, the presence of a large number of stars in the background of a star atlas geometrically increases the computational effort as the number of images and pixels increases, thereby increasing the complexity of the algorithm and the rate at which detections are missed. In response to this problem, this study proposes an improved MHT method for pinpointing stars by measuring local asymmetries within a small region surrounding each pixel in a single star atlas.Fig. 4 shows the overall flowchart of the algorithm, this method effectively removes background stars while also generating high-resolution and high-contrast images.

### Stellar removal

**Figure 5:**
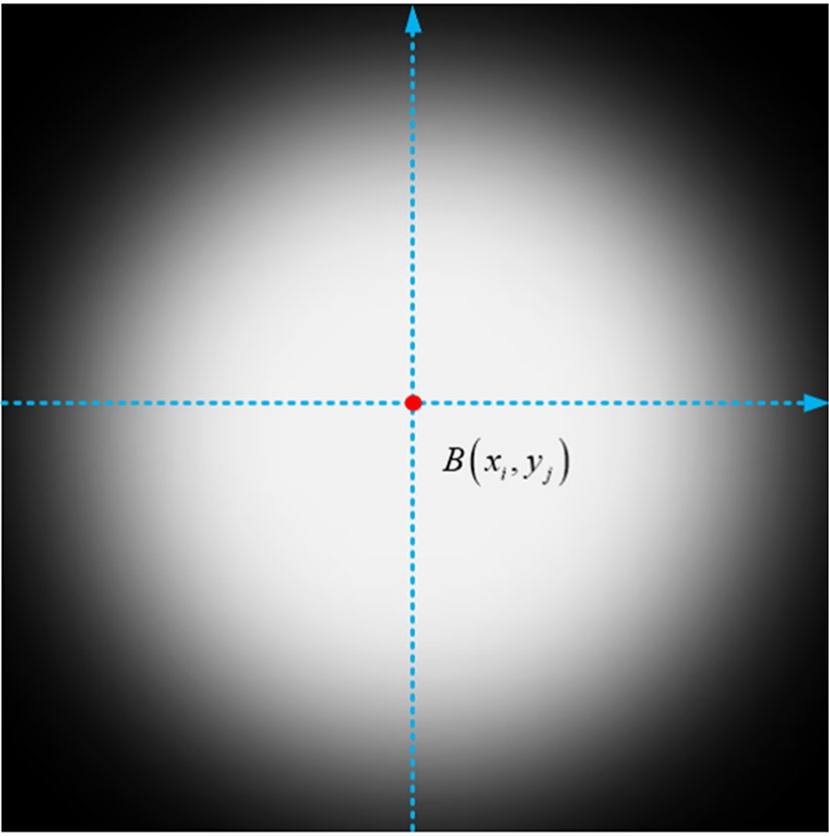
Ideal stellar point source.

As seen in Fig. 5, background stars on a star chart often form excellent point sources in images. The spread function is based on the fact that the intensity gradient differential around stars is approximately zero, and it has the form^20^

15 $$C(x,y,t) = \frac{1}{{1 + a \cdot S \cdot \exp \left( {\frac{{I_{avg} }}{{B_{avg} }} - 1} \right)}}$$

where $a$ is the asymmetry factor, $S$ is the local stop function, $\exp \left( {\frac{{I_{avg} }}{{B_{avg} }} - 1} \right)$ is the enhancement factor, $I_{avg}$ is the local average intensity, and $B_{avg}$ is the average background intensity of the whole image.

**Figure 6:**
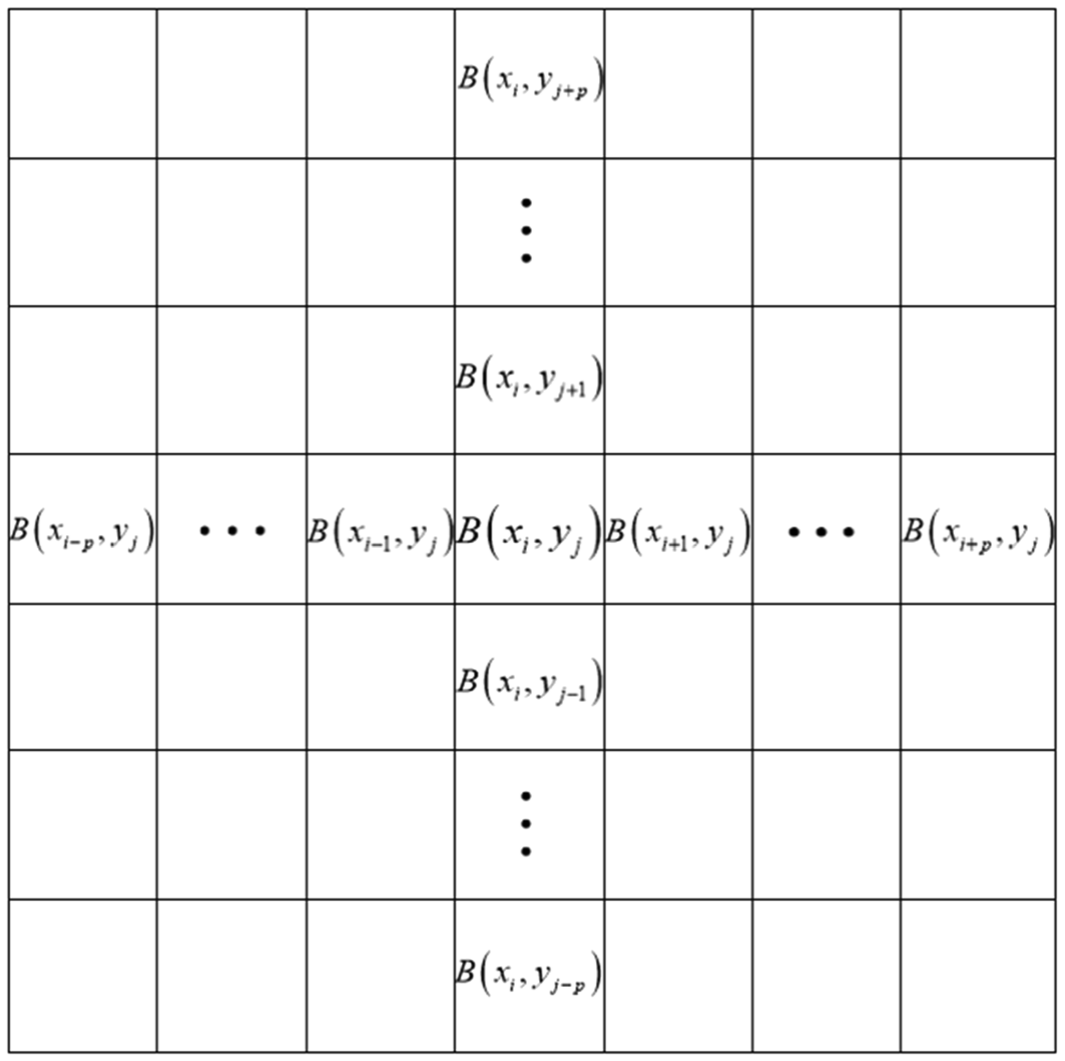
Framework for calculating the symmetry factor.

The framework for calculating the symmetry factor is shown in Fig. 6. The local symmetry of the star atlas is calculated using $a$. The diffusion function $C\left( {x,y,t} \right)$ is inversely proportional to $a$; the smaller the value of $a$, the stronger the symmetry. The diffusion function smooths symmetric regions, while regions with asymmetric edges are not affected. The formula for *a* is

16 $$a = \frac{{\sum\limits_{p = 1}^{q} {\left( {\left| {\nabla B(x_{i} ,y_{j + p} ) - \nabla B\left( {x_{i} ,y_{j - p} } \right)} \right| + \left| {\nabla B(x_{i + p} ,y_{j} ) - \nabla B\left( {x_{i - p} ,y_{j} } \right)} \right|} \right)} }}{q}$$

where *p* indicates the initial window size and *q* is the maximum value of the window.The range of *q* values usually depends on the maximum target size in the star atlas.

When the target features are smoothed, the value of S converges to $\infty$, following which the diffusion function *C* converges to 0. This ensures that the spatial target information is preserved during the processing of the star atlas. The function *S* is defined as

17 $$S = \left| {B^{n} - B^{n - 1} } \right|$$

where $B^{n}$ represents the *n*th repetition of image $B\left( {x,y} \right)$.

In the improved MHT algorithm, the diffusion function eliminates stars from the star atlas and also the suspected spatial target, thus eliminating the need to process every pixel in the star atlas. The test conditions are then assigned to form the suspected target into a tree structure based on the trajectory feature information on the motion of the target. The motion trajectory is then pruned on each node of the tree structure.

### Detection of space-based targets

Test conditions are defined according to the trajectory characteristics of the potential target points as follows:

18 $$\left\{ \begin{gathered} d - \varepsilon \le \left| {\frac{{\left| {\overrightarrow {{z_{a} z_{b} }} } \right|}}{{\left| {k_{a} - k_{b} } \right|}} - \frac{{\left| {\overrightarrow {{z_{a} z_{k} }} } \right|}}{{\left| {k - k_{b} } \right|}}} \right| \le d + \varepsilon \Rightarrow H{}_{1} \hfill \\ \begin{array}{*{20}c} {\begin{array}{*{20}c} {\begin{array}{*{20}c} {} & {} & {} & {otherwise.} \\ \end{array} } & {} \\ \end{array} } & {\begin{array}{*{20}c} {} & \Rightarrow & {H_{2} } \\ \end{array} } \\ \end{array} \hfill \\ \end{gathered} \right.$$

19 $$\left| {\overrightarrow {{z_{a} z_{b} }} } \right| = \sqrt {\left( {x_{b} - x_{a} } \right)^{2} + \left( {y_{b} - y_{a} } \right)^{2} }$$

20 $$\left| {\overrightarrow {{z_{a} z_{k} }} } \right| = \sqrt {\left( {x_{k} - x_{a} } \right)^{2} + \left( {y_{k} - y_{a} } \right)^{2} }$$

where *H*_1_ represents the candidate target point on the trajectory; *H*_2_ represents the candidate target point not on the trajectory; $z_{a}$, $z_{b}$, and $z_{k}$ are points on three distinct frames inside a frame set; their centre-of-mass coordinates are $(x_{a} ,y_{a} )$, $\left( {x_{b} ,y_{b} } \right)$, and $\left( {x_{k} ,y_{k} } \right)$; $k_{a}$, $k_{b}$, and *k* represent the frame indices (in that order); and *d* is the distance threshold (with various velocities for spatial targets at various orbital heights). The distance threshold *d* is determined adaptively based on the interframe relative velocity of the target (given that real measurements include errors) with $\varepsilon$ as the error factor.

**Figure 7:**
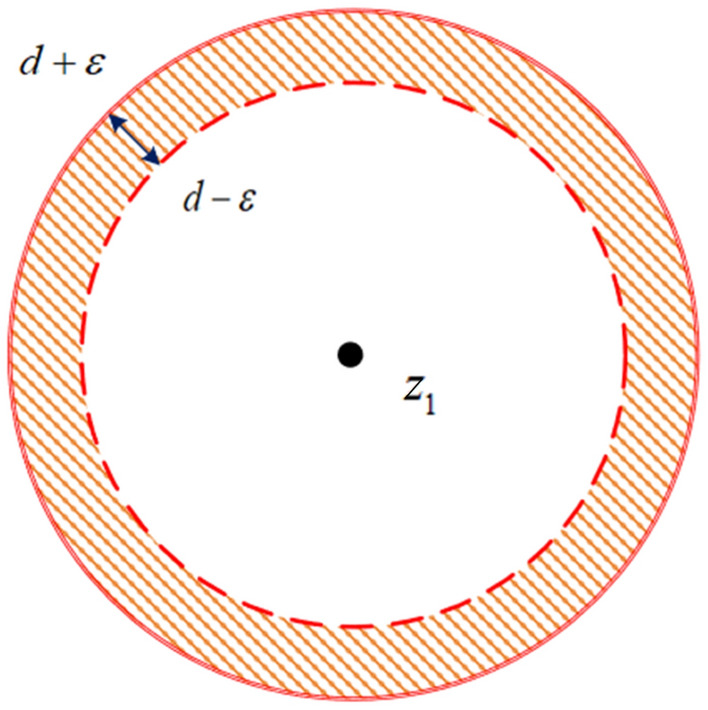
Search interval.

The variable $z_{1}$ is assigned to the presumed spatial target point in the first frame of the image sequence, and a range search is conducted using $z_{1}$ as the centre point. Figure 7 depicts the search interval. There are two types of detection results:

① If a point fulfilling the interval range cannot be identified in the search result of the second frame, the point discovered in the second frame is utilised as the new centre point for the range search.
② The search result in the second frame indicates that a point satisfying the interval range has been identified, and the point is subsequently labelled $z_{2}$. Using the centre point $z_{1}$ as the starting point, a ‘vector-to-vector’ multistage parallel discriminant tree for hypothesis testing is formed using the points in the successive frames.

The suspected spatial target sites discovered in the detection phase are integrated into candidate trajectories, which are then assessed according to

21 $$\sum\limits_{k = 1}^{K} {P_{k} } \left\{ {\begin{array}{*{20}c} { \ge D_{1} } & { \Rightarrow {\text{ true trajectory}}} \\ { \le D_{2} } & { \Rightarrow {\text{false trajectory}}} \\ { \in \left( {D_{1} ,D_{2} } \right)} & { \Rightarrow {\text{suspected trajectory}}} \\ \end{array} } \right.$$

where *K* is the number of image sequence frames necessary for a search assessment, $P_{k}$ is the *k*-point trajectory evaluation score, and $D_{1}$ and $D_{2}$ are two thresholds defined according to the value of *K* (in this study, *K* = 5, $D_{1} = 4$, and $D_{2} = 2$).

The reason for choosing $D_{1}$ and $D_{2}$ is to determine whether the detected target is a real trajectory. Simply speaking, suppose *K* = 5 means there are 5 frames of images; $D_{1}$ = 4 means there are more than 4 frames of 5 frames of images containing targets; $D_{2}$ = 2 means there are only less than 2 frames of 5 frames of images containing targets. Therefore, we can judge that $D_{1}$ belongs to real trajectory and $D_{2}$ belongs to false trajectory. In another case, when 3 frames out of 5 are detected, we cannot judge whether they are real or false trajectories, and these trajectories belong to suspected trajectories. The judgment of the suspected trajectory depends on $D_{3}$ (Eq. 22), the value of $D_{1}$ and $D_{2}$ depends on the detection accuracy of the target, the ideal detection accuracy is 100% and does not contain false alarms and clutter, then the value of $D_{1}$ = 5, $D_{2}$ = 0. In practice, in order to ensure that the detection algorithms in different environments is usually taken as the effectiveness of the $D_{1}$ = 4, $D_{2}$ = 2.

Next, the suspected track is retained, the next frame set is considered, a second assessment is conducted, and then the criteria

22 $$\sum\limits_{k = 1}^{{K_{2} }} {P_{k} \left\{ {\begin{array}{*{20}c} { \ge D_{3} } & { \Rightarrow {\text{true trajectory}}} \\ { < D_{3} } & { \Rightarrow {\text{false trajectory}}} \\ \end{array} } \right.}$$

are applied, where $K_{2} = 2 \times K = 10$ and $D_{3} = 6$.

**Figure 8:**
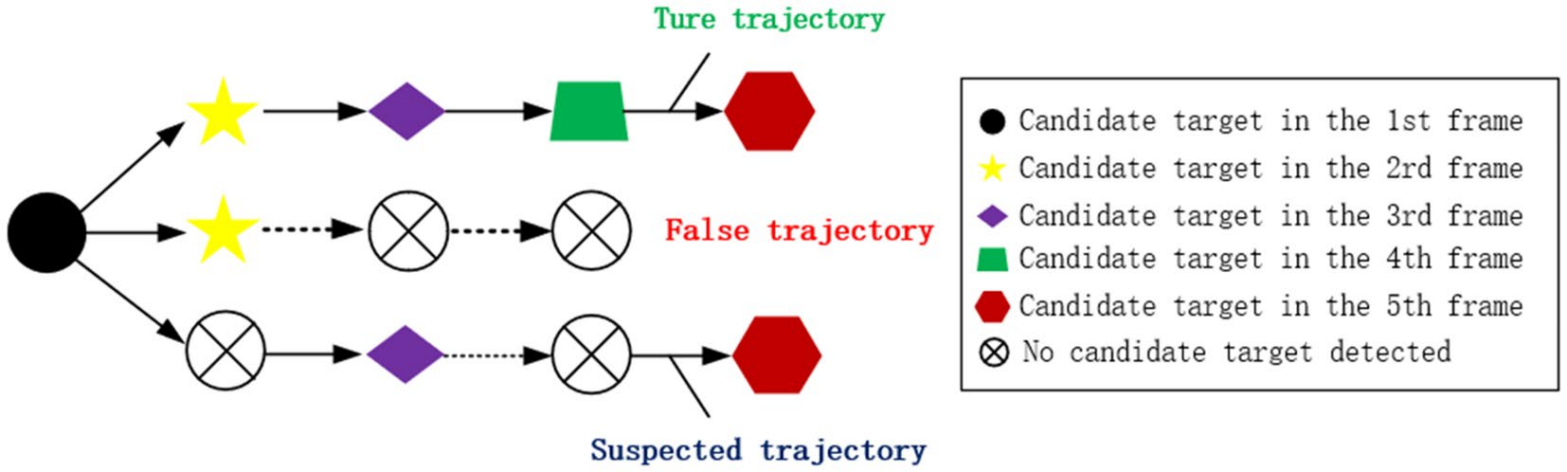
Process for differentiating between spatial target trajectories.

Figure 8 depicts the method used for differentiating between spatial target trajectories. All real trajectories are recorded and all candidate points constituting the real trajectories on each frame are retained as real target points. False trajectories are deleted and the corresponding candidate points on each frame are eliminated to finish detecting all candidate targets in the image sequence.

## Experimental evaluation

The detection method was validated using simulated and actual star atlases. The simulated star atlas was constructed by adding background stars without image shifts, spatial targets with varying streak lengths, and noise to each batch of simulated images. The actual star atlas dataset contained images captured by ground-based telescopes operating in the star tracking mode.

### Simulations of star atlas

**Figure 9:**
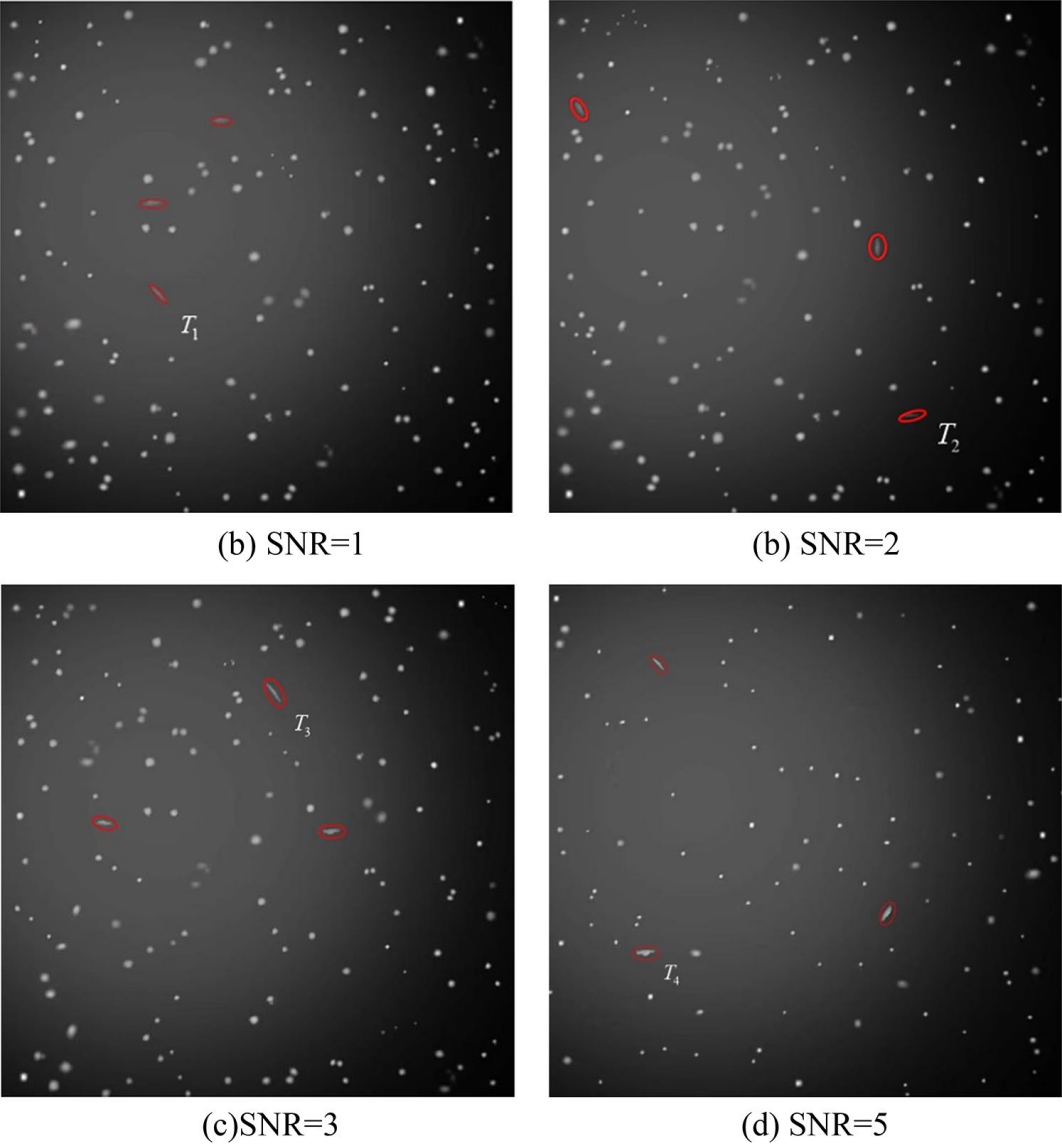
Simulation of experimental image data with various signal-to-noise ratios.

As depicted in Fig. 9, four sets of star atlases were used to conduct faint and small space-based target detection experiments. Each set of simulated images contained five consecutive frames, and the number of tested space-based targets was three. The experiments were conducted for four different target signal-to-noise ratios, and after star atlas preprocessing, the improved MHT algorithm was used to detect faint targets. The reason for choosing SNRs of 1, 2, 3, and 5 is that this manuscripts mainly focuses on the detection of dark and weak small targets. The signal strength of dark and weak small targets is relatively low, and the SNR may be lower, so the signal-to-noise ratio is chosen to be below 5.

**Figure 10:**
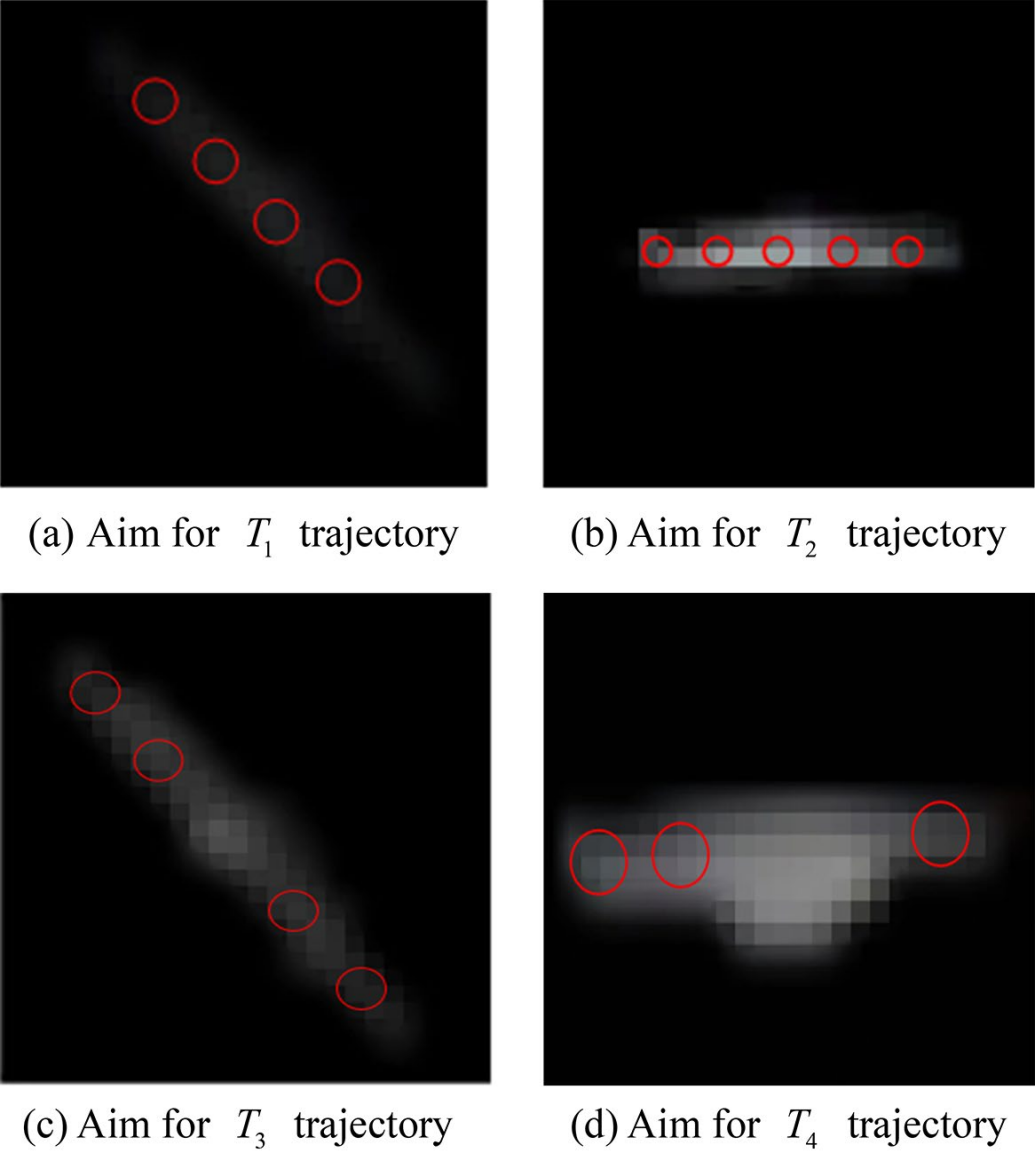
Results of spatial target trajectory identification for various signal-to-noise ratios.

Figure 10 depicts the results of the spatial target trajectory identification for various signal-to-noise ratios; the red circles in the figure represent the putative spatial target sites that are identified. The simulated image had five test frame groups; space-based targets *T*_1_ and *T*_3_ occurred in four frames of the test frame group, while *T*_2_ was correctly recognised in all five frames. The decision criteria identified *T*_1_, *T*_2_, and *T*_3_ as legitimate space-based targets. Due to occlusion by a star, the target point *T*_4_ appeared in just three frames and its potential trajectory was unknown. The succeeding findings demonstrated that the point was identified in all five frames of the subsequent test frame group and in a total of eight frames in the first and second test frame groups, indicating that the candidate point was a genuine spatial target.

Five metrics were used to measure the detection performance: detection rate $P_{d}$, false alarm rate $P_{f}$, the receiver operating characteristics (ROC) curve, the precision recall (PR) curve, and the area under the curve (AUC). The AUC is the integral of the area under the ROC curve and is a joint measurement of the detection probability and false alarm rate. The detection rate and false alarm rate are respectively calculated as

23 $$P_{d} = \frac{{N_{target} }}{{T_{target} }}$$

24 $$P_{f} = \frac{{N_{pixel} }}{{T_{pixel} }}$$

where $N_{target}$ represents the number of spatial targets discovered, $T_{target}$ represents the number of true spatial targets, $N_{pixel}$ represents the total number of false detections, and $T_{pixel}$ represents the the total number of negative pixels.

**Figure 11:**
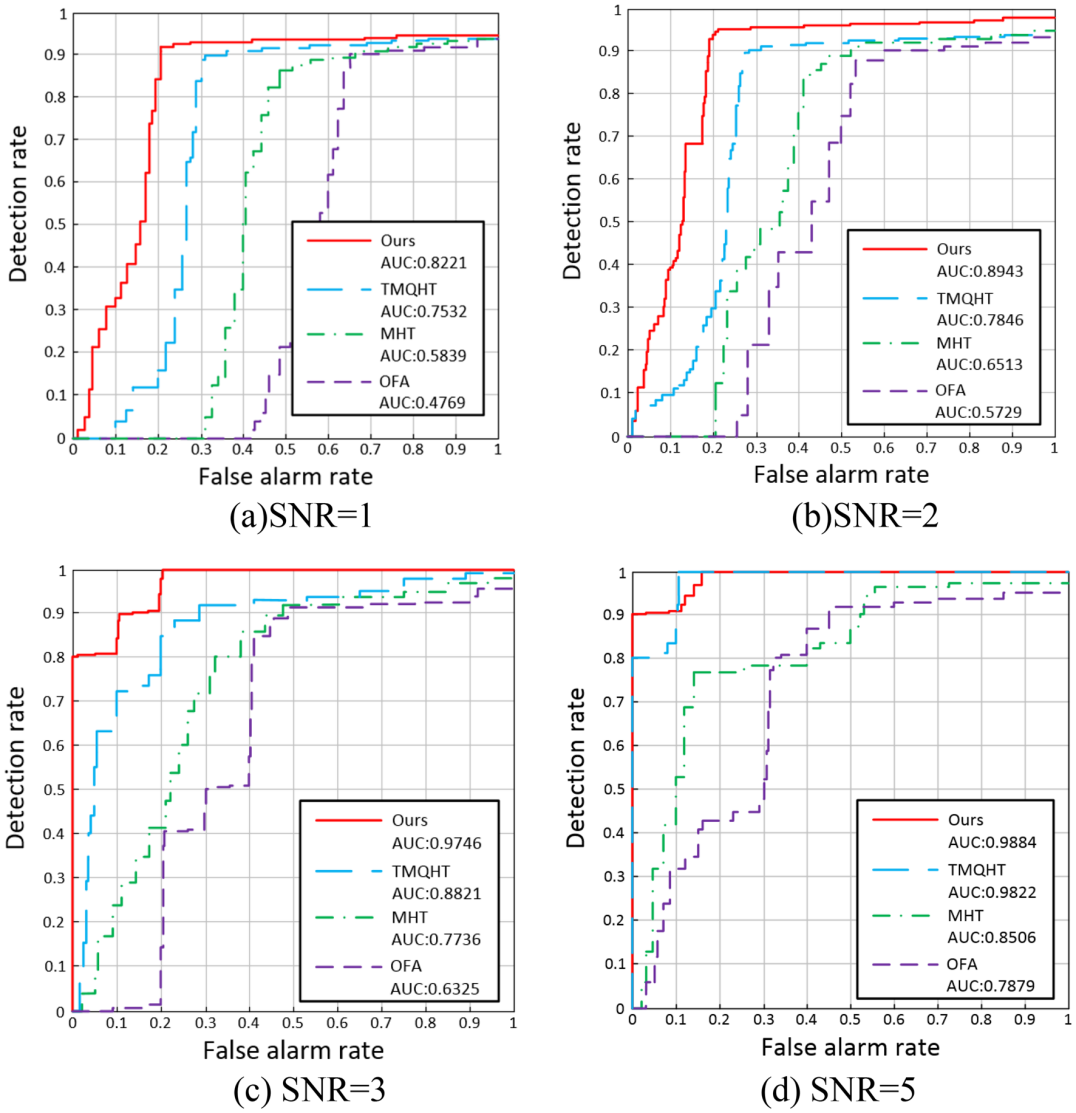
ROC curve measured by simulation data set for various SNRs.

**Figure 12:**
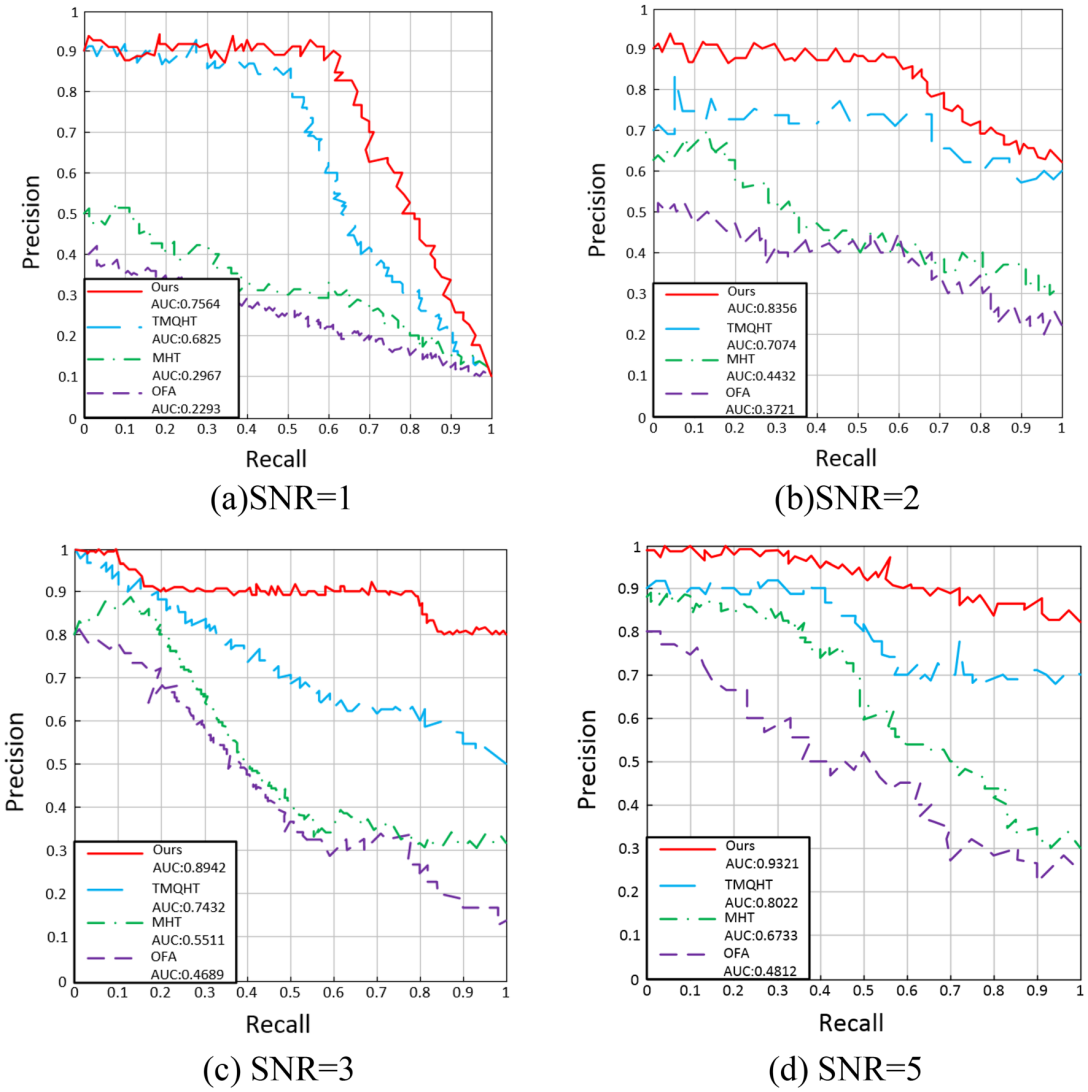
PR curve measured by simulation data set for various SNRs.

In order to validate the detection performance of the improved MHT algorithm, its performance was compared to that of the optical flow algorithm (OFA)^21^, MHT algorithm^16^, and time-index multistage quasi-hypothesis testing (TMQHT)^22^ algorithm using the same simulated image dataset. As demonstrated in Figs. 11 and 12, as the desired signal-to-noise ratio decreases, the detection rate of all four detection methods decreases. For signal-to-noise ratios of 1, 2, 3, and 5, the ROC-AUC values of the improved MHT algorithm are 82.21%, 89.43%, 97.46%, and 98.84%, respectively. The PR-AUC^23–25^ values of the improved MHT algorithm are 75.64%, 83.56%, 89.42%, and 93.21%. As demonstrated in Table 1, when the signal-to-noise ratio is greater than or equal to 3, the improved MHT algorithm can detect all spatial targets with a detection rate of 100% and a false alarm rate of 0%. At a signal-to-noise ratio of 2, the algorithm has a detection rate of 99.4% and a false alarm rate of 2.2%. For image datasets with signal-to-noise ratios between 1 and 5, the proposed technique provides superior target identification and false alarm suppression compared to the other detection algorithms.

**Table 1:** Statistical results of space-based target detection in simulated image data set.

Methods	Detection rate (%)				False alarm rate (%)			
	SNR = 5	SNR = 3	SNR = 2	SNR = 1	SNR = 5	SNR = 3	SNR = 2	SNR = 1
OFA	90.2	70	51	32	8	14.2	22.3	36.7
MHT	96.4	91.2	79	66	2.7	3.6	19.8	24.1
TMQHT	96.5	96.4	92.1	89.1	0	1.3	4.1	6.2
Ours	100	100	99.4	92.8	0	0	2.2	6.4

### Real target detection tests

**Figure 13:**
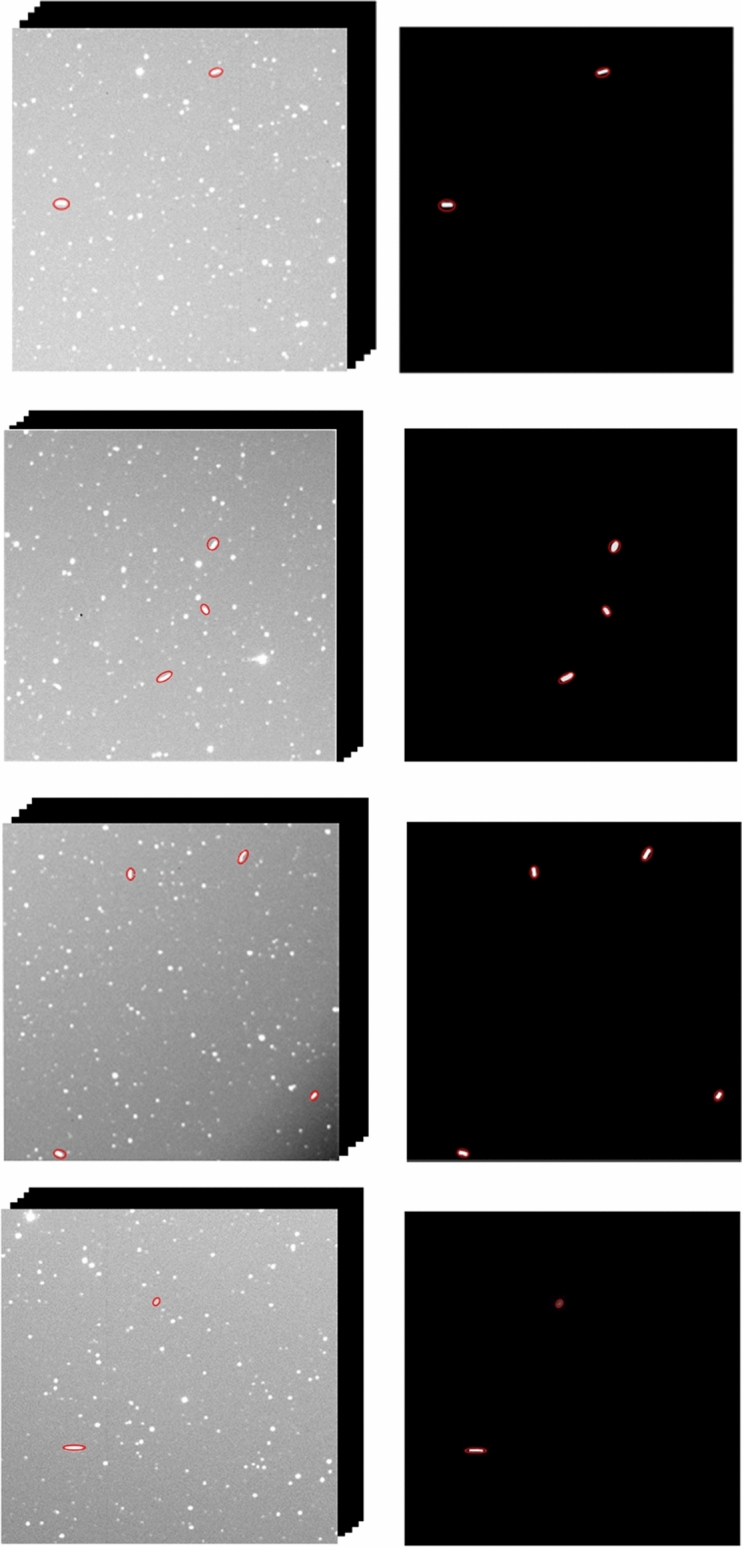
Target detection results for ground-based images.

To further validate the detection capability of the proposed algorithm, we conducted experiments on real images of stars that contained multiple spatial targets. Because live in-orbit images of space-based surveillance systems are currently unavailable, the performance of the algorithm was validated on a dataset composed of real ground-based images. To detect the differences between the target morphology, target brightness, and image background, the proposed detection algorithm primarily determines the search area of the target in the next frame based on the target motion characteristics, which prevents the morphological changes of the target from affecting the algorithm. Furthermore, when detecting the differences between the target brightness and image background, ground-based images are affected by atmospheric interference and other influences that result in lower target brightnesses and more complex background noise. Thus, the combination of ground-based images and simulated space-based images was used to verify the ability of the algorithm to detect faint and small targets and to improve the reliability of the detection results, which are shown in Fig. 13.

As shown in Table 2, the improved MHT method exhibits the best overall performance, with a detection rate of 98.6%, a false alarm rate of 4.8%, and a computation time of 0.85 s. The traditional MHT method requires every pixel in the sequence image to be processed, which necessitates a longer computation time. In contrast to the traditional MHT algorithm, the TMQHT algorithm introduces a priori information on the spatial target and prunes the MHT search tree by using the hypothesis testing conditions associated with the candidate target trajectory, thus improving the processing efficiency and the detection performance of the algorithm by a certain extent. Moreover, the OFA method requires the estimation of the trajectory information on all the targets in the image sequence, resulting in a higher computational complexity and a lower detection efficiency.

**Table 2:** Statistical results of spatial object detection in real image datasets.

Method	Detection rate (%)	False alarm rate (%)	Time (s)
OFA	88.42	10.65	12.04
MHT	90.23	16.8	26.84
TMQHT	95.24	14.2	3.25
Ours	98.6	4.8	0.85

The proposed algorithm is free from stellar interference during detection and only needs to search for the suspected spatial target, which overcomes the disadvantage of the traditional MHT algorithm in needing to search all pixel points of the image sequence, resulting in a longer computation time. Additionally, in contrast to the TMQHT algorithm, the proposed algorithm uses the relative interframe motion distance of the target to determine the area in which the suspected spatial target is located in the next frame of the image. As a result, the proposed algorithm can effectively detect spatial targets travelling at different speeds, effectively overcoming the inability of the TMQHT algorithm to detect spatial targets with changing speeds (which is caused by its application of a fixed search radius to all targets).

## Conclusion

This study proposed an improved MHT method by which space-based surveillance systems can detect faint and small space-based targets in the star tracking mode. An improved multi-exposure pyramidal weighted fusion technique was used in the image preprocessing stage to improve the overall clarity of the target image, highlight image details, and provide additional information for effective target detection. The target detection stage utilised an improved MHT method, in which a diffusion model was built to smooth the stars in the star atlas, significantly reducing the computational complexity of the algorithm and improving its detection performance. Finally, the performance of the improved MHT algorithm was verified using simulated and real star atlases. The results showed that the proposed algorithm could more efficiently detect faint and small space-based targets even under low signal-to-noise ratio conditions, as demonstrated by its higher target detection rates, lower false alarm rates, and faster computation time compared to other algorithms. Thus, the proposed method improves space object detection capability to help prevent collisions with other space objects, including important facilities such as satellites. Simultaneously, this research also provides improved technical support for space target tracking and identification. We emphasise that the method is predominantly designed for deep space target detection. For ground observation use, it has no advantage over other methods, and for cluster target detection, the method will require a long computation time but provide a reduced detection accuracy. Finally, future research could focus on integrating the proposed method with correlation filtering or multi-sensor combination methods for improved cluster target detection capability.

## Data Availability

The datasets generated and/or analysed during the current study are not publicly available due confidentiality agreement for the Space-based target photoelectric detection system in our laboratory, but are available from the corresponding author on reasonable request.
